# The Studies on α-Pinene Oxidation over the TS-1. The Influence of the Temperature, Reaction Time, Titanium and Catalyst Content

**DOI:** 10.3390/ma14247799

**Published:** 2021-12-16

**Authors:** Agnieszka Wróblewska, Jadwiga Grzeszczak, Piotr Miądlicki, Karolina Kiełbasa, Marcin Kujbida, Adrianna Kamińska, Beata Michalkiewicz

**Affiliations:** Department of Catalytic and Sorbent Materials Engineering, Faculty of Chemical Technology and Engineering, West Pomeranian University of Technology in Szczecin, Piastów Ave, 42, 71-065 Szczecin, Poland; jadwiga.tolpa@zut.edu.pl (J.G.); piotr.miadlicki@zut.edu.pl (P.M.); Karolina.Kielbasa@zut.edu.pl (K.K.); marcin.kujbida@zut.edu.pl (M.K.); kaminska.adrianna@zut.edu.pl (A.K.); Beata.Michalkiewicz@zut.edu.pl (B.M.)

**Keywords:** TS-1 catalyst, oxidation, α-pinene, α-pinene oxide, verbenol, verbenone

## Abstract

The work presents the results of studies on α-pinene oxidation over the TS-1 catalysts with different Ti content (in wt%): TS-1_1 (9.92), TS-1_2 (5.42), TS-1_3 (3.39) and TS-1_4 (3.08). No solvent was used in the oxidation studies, and molecular oxygen was used as the oxidizing agent. The effect of titanium content in the TS-1 catalyst, temperature, reaction time and amount of the catalyst in the reaction mixture on the conversion of α-pinene and the selectivities of appropriate products was investigated. It was found that it is most advantageous to carry out the process of α-pinene oxidation in the presence of the TS-1 catalyst with the titanium content of 5.42 wt% (TS-1_2), at the temperature of 85 °C, for 6 h and with the catalyst TS-1 content in the reaction mixture of 1 wt%. Under these conditions the conversion of α-pinene amounted to 34 mol%, and the selectivities of main products of α-pinene oxidation process were: α-pinene oxide (29 mol%), verbenol (15 mol%) and verbenone (12 mol%). In smaller quantities also campholenic aldehyde, trans-pinocarveol, myrtenal, myrtenol, L-carveol, carvone and 1,2-pinanediol were also formed. These products are of great practical importance in food, cosmetics, perfumery and medicine industries. Kinetic studies were also performed for the studied process.

## 1. Introduction

Titanium-silicate catalysts, both microporous (TS-1, TS-2, Ti-BETA, Ti-MWW) and mesoporous (Ti-MCM-41, Ti-MCM-48, Ti-SBA-15, Ti-SBA-16), enjoy the constant interest of researchers. The first group of catalysts mentioned above is used mainly in reactions involving small size organic molecules, while the second group has found applications in reactions involving large and branched organic molecules—it is mainly related to the pore diameter of these catalysts. The advantage of these catalysts is that they can be easily separated from the post-reaction mixture and can be used multiple times in the process. These catalysts also have disadvantages (especially those of the older generation, e.g., Ti-MCM-41 or TS-1). These disadvantages mainly relate to the stability of their structure under hydrothermal conditions, as well as leaching from their structure of titanium, which is the active center of these catalysts.

Currently, research is being carried out on new methods of synthesis these catalysts, for example by introducing an additional step of crystallization during the synthesis of these materials, with the previously obtained porous structure dissolving earlier—an example may be the hollow TS-1 catalyst (HTS-1) [1]. Two methods for the synthesis of HTS-1 have been described: acid-base treatment and base treatment. In the first method, in order to dissolve the originally obtained TS-1 porous structure, organic and inorganic acids are used (e.g., hydrochloric acid) and their salts (e.g., ammonium chloride). The acid-treated TS-1 is then added to an organic base (e.g., amine or quaternary ammonium salt). The acid treatment is omitted in the second method. Larger spaces in the HTS-1 crystal lattice are obtained using the acid-base treatment [2]. In comparison to the standard TS-1, HTS-1 has unique voids in the crystal lattice and is characterized by a high content of titanium [3]. New titanium-silicate porous materials having of micro- and mesoporous structure are also obtained based on the structure of the TS-1 catalyst and using hexadecyltrimethylammonium bromide [4]. The catalysts obtained in this way were more active in the epoxidation of cyclohexene than TS-1 catalyst [4,5]. Another and very important way of modifying of titanium-silicate catalysts and increasing their activity can be also modification of TS-1 catalyst by the increasing the amount of titanium incorporated into the structure of titanium-silicate catalysts [6].

For the first time, the TS-1 catalyst was obtained by the hydrothermal method in the early 1980s at the Enichem group research center in Italy, and it was one of the greatest achievements in catalysis [7,8,9]. This porous material is characterized by a three-dimensional system of channels with a diameter of about 0.5 nm (linear-zigzag pattern, MFI-type structure) [7]. Currently, the TS-1 catalyst is one of the most frequently used titanium-silicate catalysts in catalytic processes (it is also used in industrial processes). It is especially used in oxidation processes with the use of hydrogen peroxide as an oxidant (e.g., epoxidation of allyl alcohol [10,11], allyl chloride [12,13,14], limonene [15], diallyl ether [16,17], cyclohexene [18], or phenol hydroxylation [19,20]. Based on the previous applications of the TS-1 catalyst we decided to select this catalyst for research on the oxidation of α-pinene.

Organic raw materials of natural origin, including terpenes, are currently very popular among research laboratories and industry. This is mainly due to the fact that they are renewable resources, which are characterized by high availability and a relatively low price [21,22,23]. α-Pinene is a terpene hydrocarbon that can be obtained from various raw materials of natural origin (biomass). The advantage of these raw materials is that they are relatively easily available and renewable resources. One of the valuable sources of this compound is turpentine, which is obtained from the resin of conifers (e.g., pine) by steam distillation or gasoline extraction of pine stump. The content of α-pinene in turpentine is 60–70%. Apart from α-pinene, the main ingredients of turpentine are: β-pinene and 3-caren [24]. α-Pinene is also the main ingredient of pine oil, which can be obtained by the steam distillation of young pine shoots and needles. The amount of α-pinene in pine oil is 14–65%. In addition to α-pinene, this essential oil includes: 3-carene (0–61%), β-pinene (1–40%), limonene (0–34%), camphene (0–8%) and β-phellandrene (0–29%) [25]. α-Pinene is a very valuable compound that can be used to obtain fragrances and biologically active compounds that are used in medicine and cosmetics (camphene, limonene, isobornyl acetate, isoborneol, camphor, terpenocyclohexanols, α-terpineol, terpinyl acetate or *p*-menthol) [26]. The antibacterial [27], antifungal [28], anti-inflammatory [29] properties of α-pinene and its anticancer activity are also of great interest [27]. 

**Figure 1:**
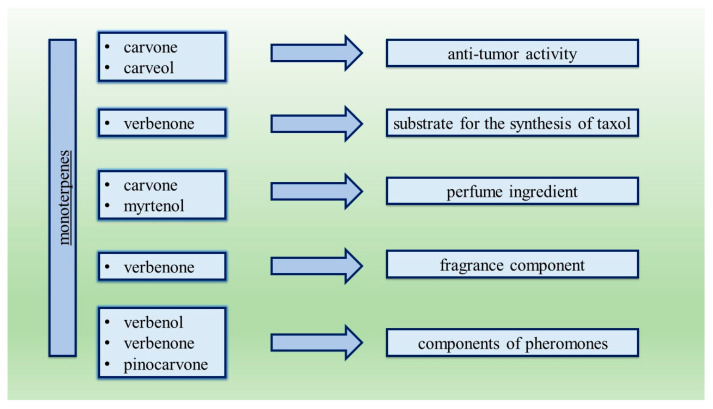
Monoterpenes obtained during oxidation of α-pinene and their applications.

In the oxidation of α-pinene, valuable oxygen derivatives of this compound can be obtained, such as: α-pinene oxide, verbenol and verbenone. The oxidation process also allows for the production of: campholenic aldehyde, trans-pinocarveol, myrtenal, myrtenol, L-carveol, carvone and pinanediol [30]. It should be emphasized that α-pinene oxidation products are very valuable compounds, widely used in medicine and in perfumery, food, and cosmetics industries. Among many compounds of natural origin, monoterpenes play the important role of both anti-cancer agents and substances used in anti-cancer therapy. For example, carvone and carveol have been shown to have anti-tumor activity in breast cancer studies [31]. Moreover, verbenone is a compound that is a substrate for the synthesis of taxol [32], a drug used in the treatment of cancer. Monoterpenes also have flavor and aroma properties that can be used in perfumery as perfume ingredients. In the perfume industry, monoterpenes obtained directly from essential oils, for example carvone and myrtenol, are of key importance [33]. Carvone is characterized by a refreshing mint aroma, and its properties make it not only used in the perfume industry, but also in the food industry and in agriculture [34,35]. Verbenone, together with verbenol, has a smell that resembles camphor and mint. These compounds are obtained from pine and eucalyptus [36]. First from these products—verbenone is used as a fragrance component in compositions with the scent of strawberry, raspberry, dill, rosemary and green mint. Moreover, the use of verbenol, verbenone and pinocarvone as components of pheromones creates new opportunities to fight bark beetles and other coniferous pests [37]. A number of other monoterpenes have been used to improve or create new, previously unknown fragrances. Attempts are being made to produce new analogues of compounds with aromatic properties, for example an analogue of santanol was obtained from (R) (−)-carvone —(Z)-normethyl-carvo-beta-santalol, with a “slightly forest smell with a hint of cedrol” [35]. Some of the monoterpenes with unique aromatic properties are formed in natural conditions by the transformation of appropriate precursors. As an example, mention should be made of (R) (+)-limonene, which is oxidized in plants of the genus Mentha, e.g., to (R) (−)-carvone (M. spicata) and (−)-menthol (M. piperita), while (S) (+)-carvone is formed by oxidation of (S) (−)-limonene in caraway fruit (Carumcarvi) [38]. All applications of products of oxidation of α-pinene are presented in Figure 1.

So far, research has been conducted with the use of heterogeneous titanium-silicalite catalysts on the oxidation of α-pinene with the participation of various oxidants, e.g., hydrogen peroxide and tert-butyl hydroperoxide. An article published in 2008 by Casuscelli et al. described the oxidation of α-pinene using the mesoporous Ti-MCM-41 catalyst. The oxidation reaction was carried out at the temperature of 70 °C for 7 h. Hydrogen peroxide was used as an oxidant for the studies. The process was carried out in acetonitrile which served as the solvent. Under the tested conditions, the following selectivity values of the main products were obtained: campholenic aldehyde up to 27 mol%, verbenol up to 18 mol%, and verbenone up to 39 mol%. When using Ti-MCM-41, the highest α-pinene conversion was achieved, equal to 37 mol% [39].

Kapoor and Raj presented the method for oxidizing α-pinene over the Ti-MCM-48 catalyst using hydrogen peroxide as the oxidant. The Ti-MCM-48 catalyst was used in an amount of 2 wt%. The reaction was carried out at 55 °C for 5 h. The product of oxidation was α-pinene oxide (selectivity 100 mol%), and the conversion of α-pinene was 8 mol% [40]. 

Suh et al. described the oxidation of α-pinene with a Ti-HMS catalyst [41,42]. The process was carried out in the presence of acetonitrile as the solvent. The oxidant was tert-butyl hydroperoxide. The reaction was carried out at the temperature of 77 °C for 24 h. Under the tested conditions, the following selectivity values of the main products were obtained: α-pinene oxide up to 5 mol%, campholenic aldehyde up to 82 mol%, verbenol up to 6 mol%, verbenone up to 51 mol%, and 1,2-pinanediol up to 5 mol% [42].

Wróblewska et al. [6] described the autoxidation of α-pinene on TS-1 catalysts with different titanium content. Originally, the α-pinene isomerization reaction was investigated. However, it turned out that instead of the isomerization reaction, the autoxidation of α-pinene takes place. Among the main products of autooxidation was determined: α-pinene oxide, verbenol, verbenone, and campholenic aldehyde. In addition, there were present also such compounds as: myrtenal, myrtenol, carveol, and 1,2-pinanediol. At the most beneficial conditions (for the catalyst TS-1 20:1): temperature 80 °C, content of the catalyst in reaction mixture 1 wt%, and reaction time 24 h, α-pinene oxide was formed with the selectivity of 30.25 mol% and at the conversion of α-pinene amounted to 41.81 mol%. 

Based on our results presented in the above-mentioned reference [6], we decided to check how the process of α-pinene oxidation would be affected by introducing the oxidant in the form of oxygen from the cylinder to the reaction environment—such studies on the oxidation of α-pinene have not been described in literature so far. Will it allow to increase the selectivity of the transformation to selected products and increase the conversion of α-pinene, which would be beneficial considering the separation of the post-reaction mixture into pure components (separation of products and their purification is very expensive with a large number of by-products)? It would also be advantageous to increase the conversion of α-pinene, then less of this organic material would have to be recycled back to the process. The aim of this work was therefore to investigate the activity of TS-1 catalyst samples with different titanium content in the oxidation of α-pinene with oxygen, but without the participation of a solvent (which would be beneficial from an ecological point of view). The research presented in this work consisted in determining the qualitative and quantitative products of α-pinene oxidation and determining the most favorable parameters of this process (temperature, reaction time and catalyst content). The aim was also kinetic studies of the tested process of α-pinene oxidation. In order to carry out research on the oxidation of α-pinene, a special installation was built to conduct these studies.

## 2. Results and Discussion

### 2.1. Characteristics of the TS-1 Materials

The titanium content in the TS-1 materials was determined by the XRF method. The results were showed in Table 1.

**Figure 2:**
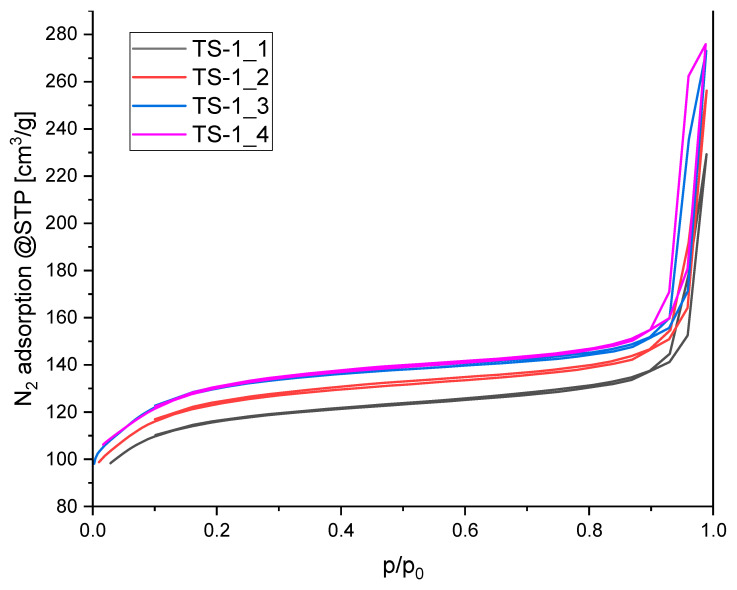
N_2_ sorption isotherms at temperature of 77 K.

The N_2_ sorption measurements at 77 K were presented at the Figure 2. 

All the isotherms were type II with H3 hysteresis representing unrestricted monolayer-multilayer adsorption. According to [43] H3 hysteresis is typical for the II isotherm and the lower limit of the desorption branch is located at the cavitation-induced p/p0. The beginning of hysteresis was located at 0.9 is usually attributed to the condensation within the voids formed between the zeolitic particles [44].

The textural properties listed in Table 1 were similar for all the TS-1 samples and typical for the TS-1 zeolite [45]. However, the influence of Ti content on the textural parameters values was clearly seen. The specific surface area, total pore volume and micropore volume decreased with titanium content. This is probably due to the existence of extra framework titanium.

**Figure 3:**
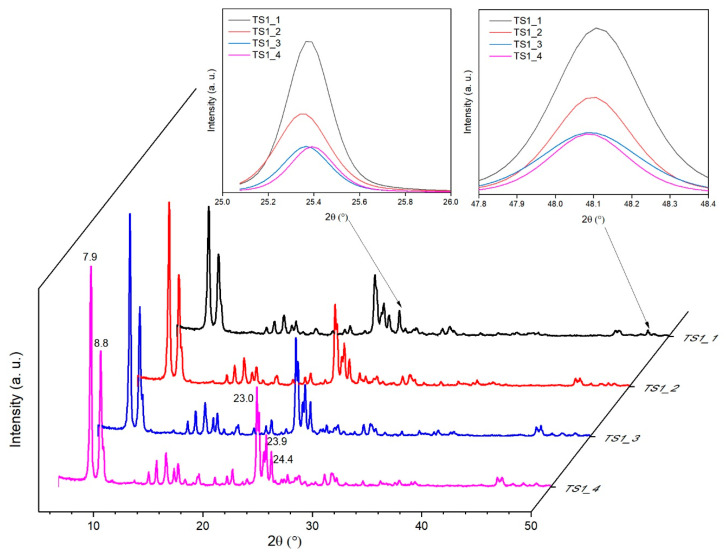
XRD patterns of the TS-1 materials.

The X-ray diffraction patterns of the TS-l are shown in Figure 3. All of the TS-1 materials had characteristic diffraction peaks of the MFI topology, that were sited at 2θ values of 7.9, 8.8, 23.0, 23.9, and 24.4° [46] proving that the addition of Ti up to about 10 wt% did not change the topology.

On the basis of single reflections around 2θ equal to 24.4° and 29.3° was stated that symmetry of the TS-l materials was orthorhombic. The orthorhombic symmetry proved the location of Ti in the zeolite framework [8]. The symmetry of pure silicate framework is monoclinic and presents double reflections at the 2θ equal to 24.4° and 29.3° [47]. The low intense peaks at 2θ = 25.4 and 48.1° characteristic of crystalline anatase (TiO_2_) were also identified [48]. These observations indicates the presence of extra-lattice Ti in TS-1 samples. The higher the concentration of titanium, the higher the intensity of these peaks and they were more visible. In order to show this phenomenon clearly, these peaks were also shown separately in the insets in the Figure 3.

**Figure 4:**
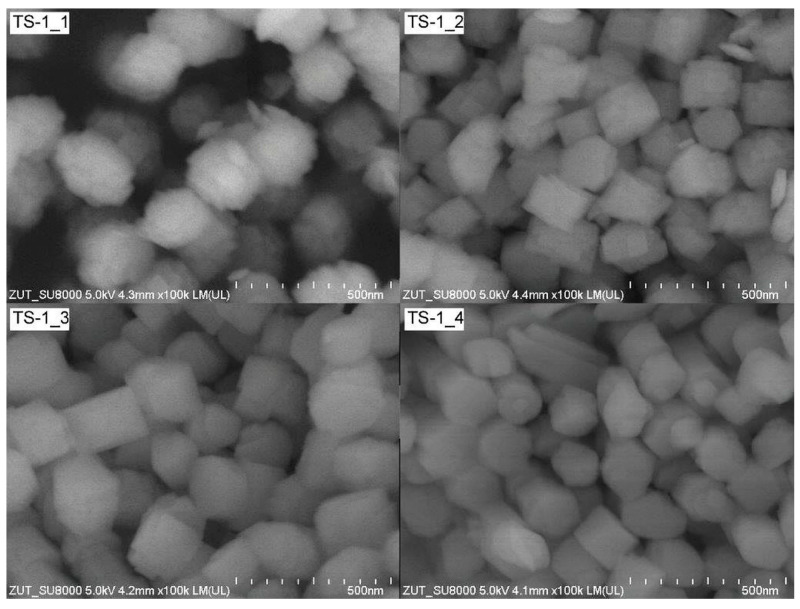
SEM pictures of TS-1 materials.

Representative SEM images of TS-1 materials were shown in Figure 4.

The SEM technique confirmed the absence of any amorphous material in all the TS-1 samples. The micrographs clearly showed that the TS-1 materials were very homogeneous, consisting of well-defined crystallites with similar hexagonal shapes. Only TS-1_1 was quite different. The hexagonal shape of it was built from the smallest crystals. The other authors found that the TS-1 crystals shapes were similar [49,50,51].

**Figure 5:**
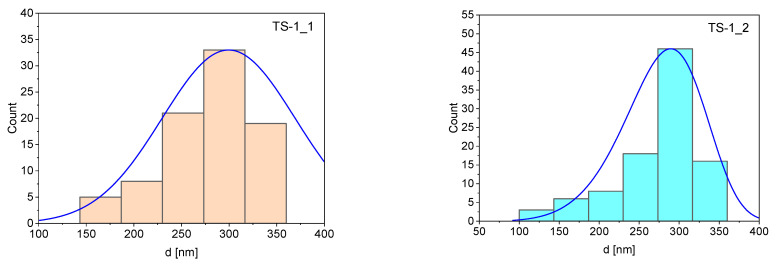

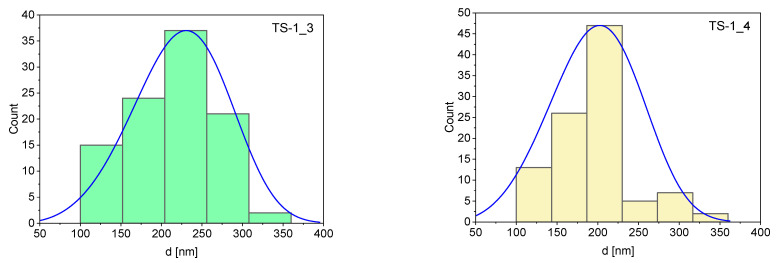
The particle-size distribution of TS-1 materials.

On the basis on SEM measurements the particle-size distribution was calculated and presented at the histogram (Figure 5). The sizes of a hundred particles were estimated for every material. The histograms showed that the sizes of particles of TS-1 materials were quite similar, and the range of the diameters was narrow. However, it is also evident that the average diameter of the TS-1_1 particles was the highest. The average diameters of the materials were in the following order: TS-1_1 > TS-1_2 > TS-1_3 > TS-1_4. It is noteworthy that the distribution profile of the diameters (blue curve) followed the Weibull function well. The average size of the particles and other statistic parameters are presented in Table 2.

### 2.2. Studies on the Catalytic Activity of TS-1 Catalysts in the α-Pinene Oxidation Process 

**Figure 6:**
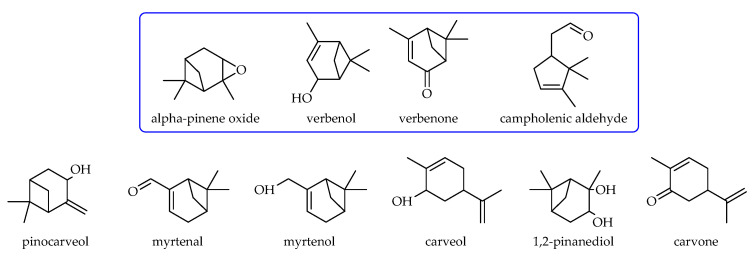
α-Pinene oxidation products.

The qualitative determinations of post-reaction mixtures made with the GC and GC-MS methods showed that the main products of α-pinene oxidation over TS-1 catalysts in the presence of oxygen as the oxidant were: α-pinene oxide, verbenol, verbenone and campholenic aldehyde (Figure 6). The following products were formed in smaller amounts: pinocarveol, myrtenal, myrtenol, carveol, 1,2-pinanediol and carvone (Figure 6). 

**Figure 7:**
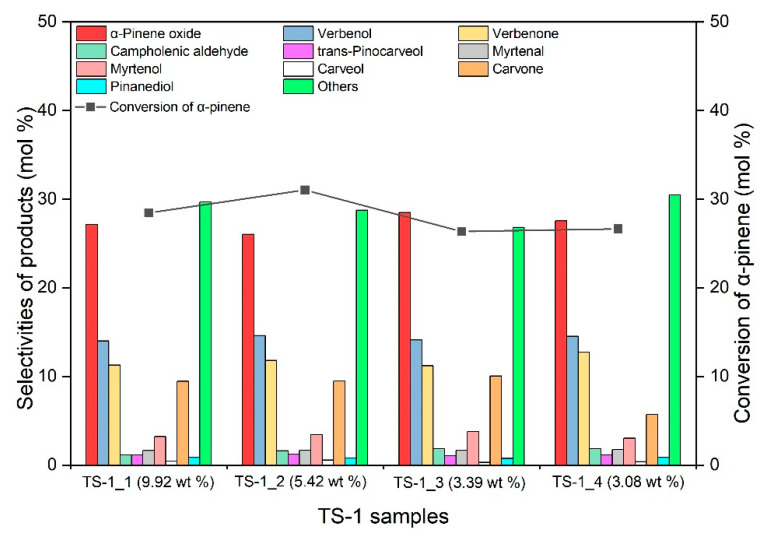
Values of conversion of α-pinene and selectivities of appropriate products in the oxidation of α-pinene over TS-1_1, TS-1_2, TS-1_3 and TS-1_4 catalysts (temperature 85 °C, catalyst content 2.5 wt% and reaction time 6 h). The figure shows the sum of the selectivities of the remaining by-products, which were formed with low selectivities.

In the first stage of the research, the influence of titanium content in the TS-1 catalyst on the conversion of α-pinene and the selectivities of the appropriate products was determined. Four catalysts with the following titanium content were used for the tests: TS-1_1 with the titanium content of 9.92 wt%, TS-1_2 with the titanium content of 5.42 wt%, TS-1_3 with the titanium content of 3.39 wt% and TS-1_4 with the titanium content of 3.08 wt%. The oxidation was carried out at the temperature of 85 °C for 6 h. The catalyst content in the reaction mixture was 2.5 wt%. The results of these studies are shown in Figure 7.

Figure 7 shows that the major oxidation product of α-pinene is α-pinene oxide. The selectivity of the transformation to α-pinene oxide remained at a similar level for the 4 tested catalyst samples and amounted to about 26–29 mol%. Similarly, the selectivities of transformation to verbenol and verbenone practically did not change during the research at this stage and amounted to approximately 15% and 11–13 mol%, respectively. Among the tested catalysts, the most active catalyst turned out to be the TS-1_2 catalyst with a titanium content of 5.42 wt%, for which the highest conversion of α-pinene, amounting to 31 mol%, was obtained. On this catalyst with the highest selectivities among the formed products, we obtained: α-pinene oxide (26.0 mol%), verbenol (14.6 mol%), verbenone (11.8 mol%) and carvone (9.5 mol%). In the case of the remaining catalysts, the α-pinene conversion was slightly lower and amounted to about 26–28 mol%. The TS-1_2 catalyst was selected as the most active for the next stage of research (temperature impact studies). Comparing the results obtained in our research with the results presented in the literature for such catalysts as carbon-supported Pt catalysts [52], it can be said that the use of TS-1 catalysts in the oxidation of α-pinene allows to obtain a lower summary selectivity of transformation to verbenol and verbenone (allylic oxidation products) than that obtained on carbon-supported Pt catalysts (respectively, about 28 mol% for all tested TS-1 catalysts and 47–59 mol% for carbon-supported Pt catalysts). In our study, the summary selectivity of the transformation to products of allylic oxidation is similar to the selectivity of the transformation to the compound formed as the result of the epoxidation (α-pinene oxide). On the other hand, the increase in selectivity of transformation to epoxy product in relation to the sum of selectivity of verbenol and verbenone was observed in studies with carbon nanotubes and activated carbons [53]. In these studies, the selectivity of the transformation to α-pinene oxide was usually about three times higher than the summary selectivity of the transformation to allylic oxidation products. Differences in the oxidation of α-pinene on our TS-1 catalysts and carbon-supported Pt catalysts, carbon nanotubes and activated carbons described in the literature may result from different structures (including the presence of pores of the appropriate volume and shape) of these catalysts and different types of active sites as well as their location in the structure of these catalysts.

**Figure 8:**
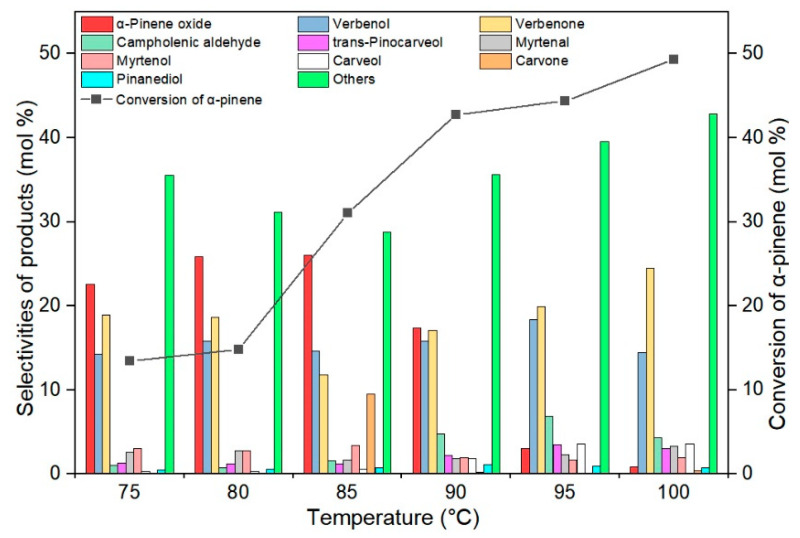
Influence of temperature on the conversion of α-pinene and selectivities of appropriate products in the oxidation of α-pinene over the TS-1_2 catalyst (catalyst content 2.5 wt%, reaction time 6 h), the figure shows the sum of the selectivities of the remaining by-products, which were formed with low selectivities.

In the second stage of the catalytic activity tests, the influence of temperature on the conversion of α-pinene and the selectivities of the appropriate products during the oxidation of α-pinene was investigated. The temperature of the oxidation process was changed in the range from 75 to 100 °C, the content of the TS-1_2 catalyst was 2.5 wt%, and the reaction time was 6 h. Changes in the conversion of α-pinene and the selectivities of the appropriate products are shown in Figure 8. 

Figure 8 shows that as the temperature of the oxidation process increases, the conversion of α-pinene increases, reaching the maximum value of 49 mol% at 100 °C. With increasing temperature, the selectivity of transformation to α-pinene oxide increases from 23 mol% (temperature 75 °C) to 26 mol% (temperature 85 °C), and then decreases to 1 mol% (temperature 100 °C). Low values of the selectivity of transformation to α-pinene oxide at temperatures in the range of 95–100 °C may indicate that it has been isomerized to campholenic aldehyde or hydrated to 1,2-pinanediol. In studies conducted at this stage, verbenol was produced with similar selectivity in the entire temperature range studied (14–18 mol%). However, with increasing temperature, the selectivity of the transformation to verbenone first decreased from 19 mol% (temperature 75 °C) to 12 mol% (temperature 85 °C), and then increased to 20 mol% (temperature 100 °C). In the studies of the influence of temperature, a very large increase in the summary selectivity of the transformation to the products of allylic oxidation in comparison to the selectivity of the transformation to the epoxy compound is visible with the increase of temperature. For the highest tested temperatures, about 7–8 times more products of allylic oxidation are obtained. The observed decrease in the selectivity of transformation to epoxide is most likely related to the low stability at higher temperatures of this epoxy compound and also its isomerization to campholenic aldehyde and carveol. The last compound can be next oxidized to carvone. Interpreting the results obtained at this stage, it was found that it is most advantageous to carry out the process of α-pinene oxidation at the temperature of 85 °C (the temperature at which the highest selectivity of the transformation to α-pinene oxide is achieved). At this temperature, the following selectivity values of the main products were obtained: α-pinene oxide 26 mol%, verbenol 15 mol%, verbenone 12 mol%, and the conversion of α-pinene was 31 mol%. 

**Figure 9:**
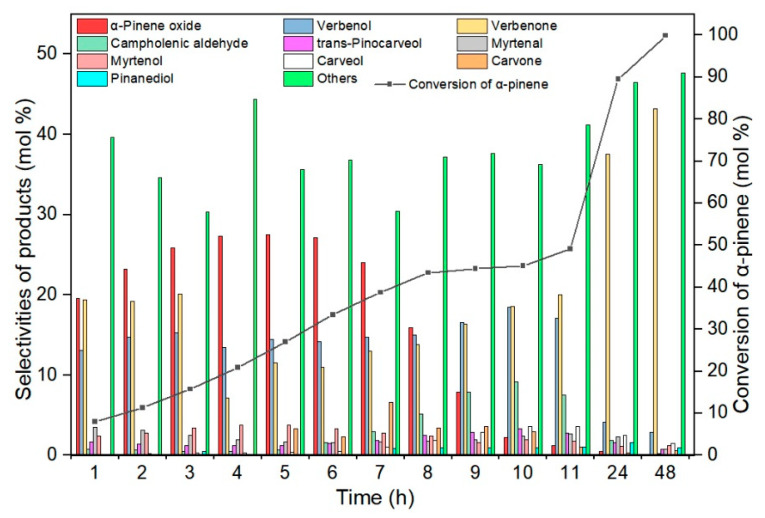
The influence of reaction time on the conversion of α-pinene and selectivities of appropriate products in the oxidation of α-pinene over the TS-1_2 catalyst (temperature 85 °C, catalyst content 2.5 wt%), the figure shows the sum of the selectivities of the remaining by-products, which were formed with low selectivities.

In order to investigate the influence of the reaction time on the conversion of α-pinene and the selectivity of the transformation to the corresponding products, a series of tests was carried out at the temperature of 85 °C, which was selected as the most favorable in the previous stage of the research. The tested range of reaction times ranged from 1 to 48 h, and the amount of TS-1_2 catalyst was 2.5 wt%. These studies showed that the longer the reaction time, the higher the α-pinene conversion, as shown in Figure 9.

Figure 9 shows that the selectivity of the transformation to α-pinene oxide increases from 20 mol% (reaction time 1 h) to 28 mol% (reaction time 5 h) and then it decreases to 1 mol% (reaction time 48 h). The selectivity of the transformation to verbenol is maintained at the level of 13–18 mol% in the range of reaction times 1–11 h, and then it decreases to 3 mol% (reaction time 48 h). With longer reaction times (24 h and 48 h) it is possible to obtain high selectivities of the transformation to verbenone (corresponding: 38 mol% and 43 mol%), while at the same time high α-pinene conversion (respectively 90 mol% and 100 mol%). In the tested range of reaction times, the conversion of α-pinene increases from 8 mol% (reaction time 1 h) to 100 mol% (reaction time 48 h). Time influence studies showed a very significant increase in the selectivity of the transformation towards allylic compounds, especially to verbenone (for the longest reaction times). The significant increase in the amount of verbenone is probably related to the conversion of verbenol to verbenone under the reaction conditions (oxidizing atmosphere). On the other hand, the reduction of the selectivity of transformation to the epoxy compound is related to the instability of this compound at elevated temperature and its isomerization to carveol and campholenic aldehyde. Research on the influence of the reaction time on the course of oxidation of α-pinene shows that the optimal results were obtained for the reaction time of 6 h (taking into account the selectivity of the transformation to α-pinene oxide), therefore this time was chosen as the most favorable for the next stage of the research. 

**Figure 10:**
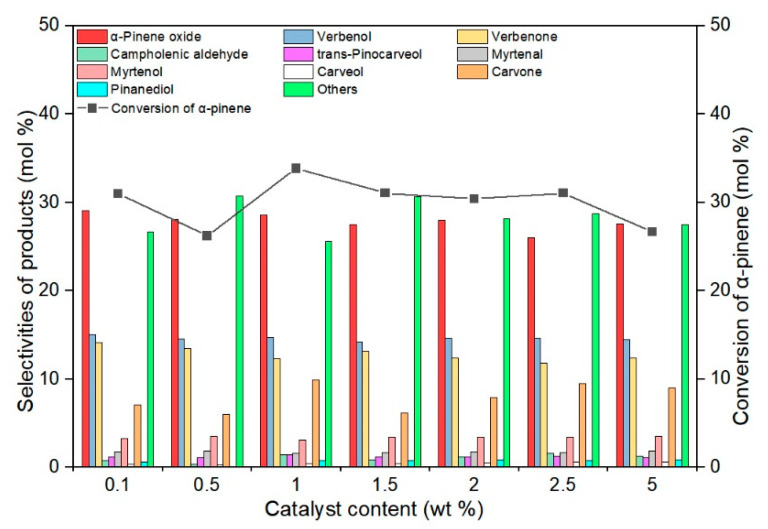
Influence of the catalyst content on the conversion of α-pinene and selectivities of appropriate products in the oxidation of α-pinene over the TS-1_2 catalyst (temperature 85 °C, reaction time 6 h), the figure shows the sum of the selectivities of the remaining by-products, which were formed with low selectivities.

The influence of the catalyst content on the conversion of α-pinene and the selectivities of the respective products was tested at the temperature of 85 °C and for the reaction time of 6 h, these parameters were considered the most favorable in the previous stages of the research. At this stage of studies, the tested range of catalyst content was from 0.1 to 5 wt%. The results of these studies are shown in Figure 10. 

Studies on the influence of the TS-1_2 catalyst content in the reaction mixture have shown that the selectivity of α-pinene oxide reaches the value of about 27 mol%, the selectivity of verbenone about 13 mol%, and the selectivity of verbenol about 15 mol%, in the entire range of the tested catalyst contents. There was also no significant influence of changes in the amount of catalyst in the reaction mixture on the selectivity of the other main products (trans-pinocarveol, myrtenal, and myrtenol). During the research at this stage, the conversion of α-pinene varied in the range of 26–34 mol%. In this case, the ratio of the selectivity of transformation to the epoxy compound and the summary selectivity of the transformation to allylic compounds was close to 1 and did not change with the increase in the amount of catalyst in the reaction mixture. The analysis of the obtained results showed that the most advantageous amount of the TS-1_2 catalyst in the reaction mixture was 1 wt% (the selection was made on the basis of the values of conversion of α-pinene and the selectivity of α-pinene oxide).

The overall kinetic studies of the α-pinene oxidation over TS-1 catalyst were also performed. The kinetic studies were modeling considering a constant oxygen uptake (expressed in mol/dm^3^) based on a series of experiments in which the effect of the different factors on the reaction rate were checked, e.g., temperature, reaction time, and amount of the catalyst. For each experiment (75, 80, 85, 90, 95 and 100 °C; P_O2_ = 1 bar) the reaction mixture composition was determined at the varied points of oxygen uptake/α-pinene ratio (defined in mol_O2__._ mol_α-pinene_). The α-pinene oxidation rates were calculated by kinetic curves differentiation. The turbulence created around the catalyst particles by a dynamic stirring of the reaction mixture helps to eliminate the external diffusion resistance between the bulk liquid and surface of the catalyst. Internal diffusion resistance was also negligible because of the small size of catalyst particles (0.24 μm ≤ dp ≤ 0.27 μm) used in the runs. It was observed that product content slightly depends on oxygen uptake/α-pinene ratio. Moreover, the α-pinene oxidation rate increases along with increase of the oxidation temperature. Activation energy estimated from Arrhenius dependence was 80.6 ± 4.7 kJ/mol and the effective kinetic constant was k_eff_ = 1.0 × 10^10^ mol^0.5^·dm^−1.5^·min^−1^ (the regression coefficient equals 0.9502). Consequently, under the reaction conditions the reaction rate of α-pinene oxidation by molecular oxygen can be expressed as follows:

R_O_ = k_eff_ f(concentration)^0.5^·P^0^_O2_·exp(−E_a_/RT) [mol·dm^−3^·min^−1^]. (1)

Calculated activation energy matches results achieved for typical values of activation energy for α-pinene oxidation by molecular oxygen (81.3 kJ/mol) [54] as well as cis-pinene oxidation (79.5 kJ/mol) [55] or dibenzyl ester oxidation initiated by azoisobutyronitrile (93.66 kJ/mol) [56].

## 3. Materials and Methods

### 3.1. Raw Materials

In the tested process, the following reagents were used for oxidation carried out with the use of titanium-silicate TS-1 catalysts: α-pinene (98%, Sigma Aldrich, Poznań, Poland), and oxygen (99.99%, Messer, Szczecin, Poland). Moreover, the following compounds were used as standards for chromatographic analyzes: α-pinene oxide (97%, Sigma Aldrich, Poznań, Poland), verbenol (95%, Sigma Aldrich, Poznań, Poland), verbenone (≥93%, Sigma Aldrich, Poznań, Poland), *trans*-pinocarveol (≥96%, Sigma Aldrich, Poznań, Poland), myrtenal (98%, Sigma Aldrich, Poznań, Poland), myrtenol (95%, Sigma Aldrich, Poznań, Poland), carveol (≥95%, Sigma Aldrich, Poznań, Poland), carvone (98%, Sigma Aldrich, Poznań, Poland), pinanediol (99%, Sigma Aldrich, Poznań, Poland). Campholenic aldehyde was identified by GC-MS method.

### 3.2. Synthesis of TS-1 Catalysts

The TS-1 catalysts were obtained by the hydrothermal method, with different molar ratios of silicon to titanium in the crystallization gel, according to the method described in the literature [6]. The obtained catalysts were marked as: TS-1_1 (titanium content 9.92 wt%), TS-1_2 (titanium content 5.42 wt%), TS-1_3 (titanium content 3.39 wt%), TS-1_4 (titanium content 3.08 wt%).

### 3.3. Characteristics of the TS-1 Catalysts

Characterization of the obtained four TS-1 catalysts was performed based on the following instrumental methods: XRF, N_2_ sorption measurements at 77K, XRD and SEM. 

The titanium content in TS-1 materials was estimated using an energy dispersive X-ray fluorescence (EDXRF) spectrometer with the Epsilon 3 PANalytical B.V. instrument (manufacturer, city, state, country). 

The surface area, total pore volume, micropore volume and pore size distribution of the TS-1 materials were determined on an automatic N_2_ sorption instrument Quadrasorb evo™ Gas Sorption analyzer (Anton Paar, St Albans, UK; previously Quantachrome Instruments, USA, 2014) at 77 K. The specific surface areas (SBET) of the samples were calculated by the Brunauer–Emmett–Teller method. The total pore volume (Vtot) was calculated utilizing the volume of N_2_ adsorption at p/p_0_ = 0.99. The t-Plot method was utilized to determine the micropore volume. The weight of the samples was in the range: 0.2708–0.3201 g. Before the measurement, the samples were dried at 250 °C for 20 h.

The XRD analysis was performed at ambient temperature using a X’Pert–PRO, Panalytical, Almelo, The Netherlands, 2012) with Cu-Kα (λ = 1.54056Å) radiation. The data were taken for the 2θ range of 5° to 50° with a step of 0.026°. The XRD patterns were compared with the JCPDS (Joint Committee on Powder Diffraction Standard) to identify the phases.

The SEM pictures were obtained using ultra-high resolution field emission scanning electron microscope (UHR FE-SEM) via the Hitachi SU8020 (Ultra-High Resolution Field Emission Scanning Electron Microscope; Hitachi Ltd, Tokyo, Japan, 2012). TS-1 materials were spread on the carbon tape and placed over the surface of SEM stub. 

### 3.4. Oxidation of α-Pinene

**Figure 11:**
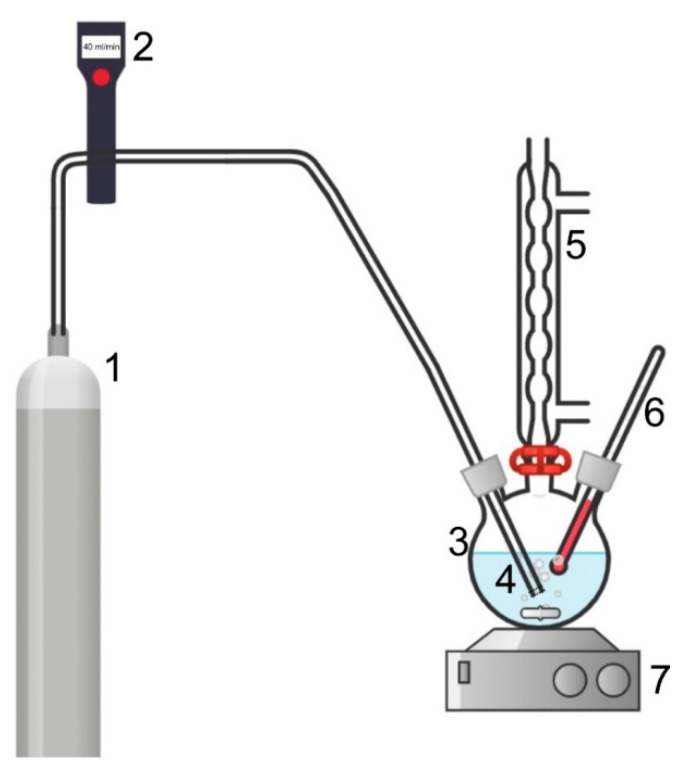
Apparatus used in the oxidation of α-pinene with oxygen: 1—oxygen cylinder, 2—oxygen flow regulator, 3—glass reactor with the capacity of 50 cm^3^, 4—glass bubbler for oxygen supply, 5—reflux condenser, 6—thermometer, 7—magnetic stirrer with heating function.

The oxidation of α-pinene over TS-1 catalysts with the use of molecular oxygen as the oxidant was carried out in a 50 cm^3^ glass reactor equipped with a reflux condenser, a magnetic stirrer with heating function and a glass bubbler for oxygen supply from the cylinder. α-Pinene was introduced into the reactor first, followed by the addition of the appropriate amount of TS-1 catalyst and the supply of oxygen from a bottle at a rate of 40 mL/min. The flask was placed in the paw, immersed in the oil bath and agitated at 500 rpm. The oxidation was carried out at the temperatures of 75–100 °C, during 1–48 h and with the catalyst content of 0.1–5 wt%. After the reaction, the post-reaction solution was separated from the catalyst using a centrifuge. Figure 11 shows the apparatus used in the oxidation of α-pinene with oxygen. 

### 3.5. Identification of the Products of Oxidation by the Gas Chromatography Method 

Qualitative and quantitative analyzes of post-reaction mixtures were carried out by means of gas chromatography using a Thermo FOCUS apparatus (Thermo Fisher Scientific, Waltham, MA, USA, 2009) with an RTX-1701 column equipped with a flame ionization detector. The analysis conditions were as follows: isothermally at 50 °C for 2 min, temperature increase at 6 °C/min to 120 °C, isothermally at 120 °C for 4 min, then temperature increase at 15 °C/min up to 240 °C, the temperature of the dispenser 200 °C, and the flow of the carrier gas 1.2 mL/min. The quantitative analysis was carried out using the method of internal normalization (the error related to chromatographic analyses was 1%). For each of the performed syntheses, a mass balance was prepared, taking into account such functions of the process as: conversion of α-pinene and selectivities of the respective products. 

Qualitative analyzes of the products present in the post-reaction mixtures were also carried out by GC-MS using a ThermoQuest apparatus equipped with a Voyager detector and a DB-5 column (filled with phenyl-methylsiloxanes with dimensions 30 m × 0.25 mm × 0.5 μm). The conditions in which the analyses were carried out were as follows: helium flow 1 mL/min, sample chamber temperature 200 °C, detector temperature 250 °C, oven temperature-isothermally for 2.5 min at 50 °C, followed by an increase at a rate of 10 °C/min up to the temperature of 300 °C. 

Before performing the analyzes by GC-MS and GC methods, the post-reaction solution was separated from the catalyst by centrifugation in a laboratory centrifuge. The solution thus separated was then diluted with acetone (0.750 mL of acetone was added to 0.250 mL of the solution).

## 4. Conclusions

The influence of titanium content on the textural properties was proved. The higher Ti content the lower values of the textural properties was observed. At the same time, along with the increase in titanium content, the catalytic activity of the obtained TS-1 catalysts should increase, because titanium is the active center of the catalyst in which the reaction we are studying takes place. Based on the catalytic tests of four titanium-silicate catalysts, it was confirmed that TS-1 catalysts are active in the oxidation of α-pinene with oxygen. During the tests it was shown that the most active catalyst in this process was the TS-1_2 catalyst, which contained 5.42 wt% of Ti. All the tested process parameters (temperature, reaction time and amount of the catalyst) influenced on the conversion of α-pinene and the selectivity of the obtained products. Increasing the temperature and extending the reaction time increased the conversion of α-pinene. It has been found that the most advantageous oxidation of α-pinene is carried out at the temperature of 85 °C for 6 h using 1 wt% of the TS-1_2 catalyst in the reaction mixture. Comparing the results presented in our earlier publication [6] with those described in this paper, it was observed that increasing the temperature from 80 to 85 °C and the use of the TS-1_2 catalyst in the amount of 2.5 wt% in the reaction mixture allows to increase the conversion of α-pinene almost doubled (up to 31 mol% from 17 mol%) compared to the studies described in our earlier work. However, this did not affect the value of the selectivity of the transformation to α-pinene oxide, as in both studies it remained at a similar level and amounted to approximately 30 mol%. Unexpectedly, in the research presented in this study, it turned out that with longer reaction times (24 and 48 h) it is possible to obtain high selectivities of verbenone (38 mol% and 43 mol%) with a very high conversion of α-pinene (90 and 100 mol%). In general, the presented studies have shown that increasing the temperature and lengthening the reaction time reduces the selectivity of peroxide compound formation, which either decomposes because its structure is not stable or isomerizes to carveol and campholenic aldehyde. The increase in the content of verbenone along with the extension of the reaction time may, however, be related to the oxidation of verbenol to verbenone under the reaction conditions (oxidizing atmosphere). 

The comparison the results obtained in our research with the results described in the literature for carbon-supported Pt catalysts [52] showed that the use of TS-1 catalysts in the oxidation of α-pinene allows to obtain a lower summary selectivity of transformation to verbenol and verbenone (allylic oxidation products) than on carbon-supported Pt catalysts and in our study (in first stage of our studies). It was changed during raising temperature and prolonging of the reaction time over chosen TS-1 catalyst (at this stage of studies the summary selectivity of transformation to verbenol and verbenone raises). The increase in selectivity of transformation to epoxy product in relation to the sum of selectivity of verbenol and verbenone was observed in studies with carbon nanotubes and activated carbons [53]. In these studies, the selectivity of the transformation to α-pinene oxide was usually about three times higher than the summary selectivity of the transformation to allylic oxidation products. In the case of our research, such significantly higher values of epoxide selectivity were not achieved, the maximum ratio of epoxy selectivity to the sum of selectivity of compounds formed as a result of oxidation at the allylic position was about 1. Differences in the oxidation of α-pinene on our TS-1 catalysts and carbon-supported Pt catalysts, carbon nanotubes and activated carbons described in the literature may result from different structures (including the presence of pores of the appropriate volume and shape) of these catalysts and different types of active sites as well as their location in the structure of these catalysts.

The main advantage of the presented process of oxidation of α-pinene with oxygen is the lack of a solvent in the reaction medium. The proposed method of transforming α-pinene in the direction of obtaining valuable compounds is an example of an environmentally friendly process with the possibility of using waste in the form of turpentine (a renewable and vegetable source of α-pinene). It is also worth emphasizing that the products of α-pinene oxidation are products with a very high application potential in medicine, as well as in the cosmetics, perfumery and food industries.

## Figures and Tables

**Table 1:** The Ti content and textural parameters of the TS-1 materials.

TS-1 Material	Ti (wt%)	S_BET_ (m^2^/g)	V_tot_ (cm^3^/g)	V_mic_ (cm^3^/g)
TS-1_1	9.92	438	0.355	0.168
TS-1_2	5.42	463	0.397	0.180
TS-1_3	3.39	488	0.423	0.191
TS-1_4	3.08	484	0.430	0.189

**Table 2:** The statistical data concerning the particle size distribution.

Sample	Mean (nm)	Standard Deviation	Minimum (nm)	Maximum (nm)
TS-1_1	299	69.8	150	450
TS-1_2	277	52.1	130	380
TS-1_3	223	58.5	110	360
TS-1_4	198	50.3	120	350
